# Classifying Vulnerability to Sleep Deprivation Using Resting-State Functional MRI Graph Theory Metrics

**DOI:** 10.3389/fnins.2021.660365

**Published:** 2021-06-07

**Authors:** Yongqiang Xu, Ping Yu, Jianmin Zheng, Chen Wang, Tian Hu, Qi Yang, Ziliang Xu, Fan Guo, Xing Tang, Fang Ren, Yuanqiang Zhu

**Affiliations:** ^1^Department of Radiology, Xijing Hospital, Fourth Military Medical University, Xi’an, China; ^2^Affiliated Wuhan Mental Health Center, Tongji Medical College, Huazhong University of Science and Technology, Wuhan, China; ^3^Department of Radiology, Yan’an University Affiliated Hospital, Yan’an, China; ^4^Department of Radiology, Affiliated Hospital of Shaanxi University of Traditional Chinese Medicine, Xianyang, China

**Keywords:** sleep deprivation, vulnerability, functional magnetic resonance imaging, machine learning, psychomotor vigilance task

## Abstract

Sleep deprivation (SD) has become very common in contemporary society, where people work around the clock. SD-induced cognitive deficits show large inter-individual differences and are trait-like with known neural correlates. However, few studies have used neuroimaging to predict vulnerability to SD. Here, resting state functional magnetic resonance imaging (fMRI) data and psychomotor vigilance task (PVT) data were collected from 60 healthy subjects after resting wakefulness and after one night of SD. The number of PVT lapses was then used to classify participants on the basis of whether they were vulnerable or resilient to SD. We explored the viability of graph-theory-based degree centrality to accurately classify vulnerability to SD. Compared with during resting wakefulness, widespread changes in degree centrality (DC) were found after SD, indicating significant reorganization of sleep homeostasis with respect to activity in resting state brain network architecture. Support vector machine (SVM) analysis using leave-one-out cross-validation achieved a correct classification rate of 84.75% [sensitivity 82.76%, specificity 86.67%, and area under the receiver operating characteristic curve (AUC) 0.94] for differentiating vulnerable subjects from resilient subjects. Brain areas that contributed most to the classification model were mainly located within the sensorimotor network, default mode network, and thalamus. Furthermore, we found a significantly negative correlation between changes in PVT lapses and DC in the thalamus after SD. These findings suggest that resting-state network measures combined with a machine learning algorithm could have broad potential applications in screening vulnerability to SD.

## Introduction

Cognitive ability and healthy brain function rely on sufficient sleep, during which metabolic waste products are cleared away (Xie et al., 2013; Fultz et al., 2019). Lack of sleep, however, can impact nearly all aspects of cognitive and emotional function, including attention, working memory, and affect (Durmer and Dinges, 2005). Notably, functional magnetic resonance imaging (fMRI) studies measuring blood oxygen level–dependent (BOLD) signals have demonstrated that sleep deprivation (SD) is associated with widespread brain network alterations (Chee and Chuah, 2008). These include changes in interhemispheric connectivity (Zhu et al., 2016, 2020), connectivity between the thalamus and prefrontal cortex (Shao et al., 2013), and compromised anti-correlation between the Default Mode Network (DMN) and dorsal attention network (Kaufmann et al., 2016).

While cognitive deficits have been well documented and reliably related to SD, large inter-individual differences in cognitive deterioration after SD have been noted (Hudson et al., 2020). For some cognitive domains, such as sustained attention, SD-induced differences in performance are stable within a given individual even when assessed months or years apart (Rupp et al., 2012). Previous studies have indicated that differences in the vulnerability/resistance of individuals to SD-induced deficits in cognition and performance are trait-like (Van Dongen et al., 2004). Thus, the underlying mechanisms of these individual differences are a current research focus. For instance, many recent brain imaging studies have attempted to identify the neural correlates of vulnerability/resistance to SD. Using the hierarchical regression model, our previous study found that the white matter integrity of the upper longitudinal tract fibers connecting the frontal and parietal lobes was negatively associated with individual differences in psychomotor vigilance task (PVT) performance after SD (Zhu et al., 2017). Another study found that stronger anti-correlations among several networks (such as between DMN and Attention networks) during rested wakefulness could predict the vulnerability of PVT performance during SD (Yeo et al., 2015).

However, as traditional approaches are based on average estimates of differences at the group level, a reliable predictive marker of cognitive vulnerability to SD has been elusive. The translational applicability of such data to clinical practice should be based on inferences at the individual rather than group level. With recent advancements in the field of machine learning, such as the support vector machine (SVM) model, a multivariate pattern recognition machine learning (ML) technique especially well-suited for discriminating high-dimensional rsFC fMRI data, measurements derived from fMRI combined with artificial intelligence algorithms have led to improvements in diagnoses, classification, and treatment outcome prediction for a range of situations (Zhao et al., 2018; Liu et al., 2020). Furthermore, multivariate machine learning techniques are more sensitive to differences that are subtle and spatially distributed because they consider inter-regional correlations, which might be undetectable using group comparisons (Liu et al., 2020). Because SD is associated with widespread changes in functional networks, graph-based measurements of network organization, such as degree centrality (DC) (Wang et al., 2011), might have potential in predicting vulnerability to SD-induced deficits in function.

In the current study, we adopted supervised machine learning-based SVM algorithms to investigate whether baseline resting wakefulness (RW) DC measures could predict inter-individual differences in PVT lapses after SD. We hypothesized that the baseline DC in hub regions of the DMN, frontal-parietal network, and thalamus could be used to accurately classify participants as vulnerable or resistant to SD.

## Materials and Methods

### Subjects

This study was approved by the clinical trial ethics committee of Xijing Hospital at the Air Force Medical University. Written informed consent was obtained from each subject prior to the study. All participants were recruited via advertisements distributed in the local community. The exclusion criteria were as follows: (1) having a history of alcohol or drug abuse; (2) having a history of psychiatric or neurological illness; (3) sleep disorders; (4) sleep later than 24 o’clock or get up earlier than 5 o’clock; and (5) claustrophobia. The Pittsburgh sleep quality index (PSQI) was used to evaluate sleeping quality (Guo et al., 2016), and subjects who scored more than five points on the PSQI test were also excluded. Hence, the final sample comprised 60 participants.

### Study Procedure

All subjects were asked to make three visits to the laboratory. During the first visit, they were briefed about the study protocol and signed the informed consent form. All subjects agreed to undergo an MRI scan after normal sleep and after 24 h of SD, which occurred on the last two visits to the laboratory. To minimize the influence of the scanning sequence on the experimental results, the experimental condition in the last two visits was presented in a pseudo-random order. The interval between these two visits was at least 1 week. The SD process began at 8:00 AM on 1 day and ended at 8:00 AM on the following day. During SD, the participants could read books or use their mobile phones. The SD took place in a room with standard light (340 lux) and the temperature was maintained at approximately 23°C. No snack food was given after midnight. The entire SD process was monitored by two researchers to prevent the subjects from falling asleep. All of the MRI scans were scheduled between 8:00 AM and 10:00 AM.

### Psychomotor Vigilance Task

A 10-min PVT was used in the current study (Basner and Dinges, 2011). The PVT task was rendered using E-prime (version 3.0) software. During the task, participants were asked to focus on a blank box in the middle of a computer screen. A millisecond counter then began to scroll at a random interval of 2–10 s. The participants were required to press the space bar to stop the counter as quickly as possible. Reaction time was displayed for 1 s as feedback so that the participants could monitor their performance. Reaction times longer than 500 ms were recorded as a lapse in performance (Zhu et al., 2017). The participants completed 10 min of the PVT every hour from 8:00 PM to 6:00 AM.

### MRI Data Acquisition

MRI data were collected using a GE Discovery MR750 3.0T scanner with a standard 8-channel head coil at Xijing Hospital. The subjects were instructed to lie quietly on the scan flatbed, wear earplugs, open their eyes, stay awake, and try to avoid sleeping (Song et al., 2020). Cotton pads and tape were used to minimize head motion. During each scan, the subjects were reminded via a microphone to stay awake, and the heart and respiratory rates of the subjects were recorded. Resting-state functional images were collected via an axial gradient-echo EPI sequence with the following parameters: TR/TE: 2,000/30 ms, FOV: 240 × 240 mm^2^, matrix size: 128 × 128, slices: 45, and a total of 210 volumes. The structural MRI data were obtained using a sagittal 3D Bravo T1-weighted scan sequence with the following parameters: TR/TE: 8.2/3.2 ms, FOV: 256 × 256 mm^2^, matrix: 256 × 256, slice thickness: 1.0 mm, slices: 196.

### MRI Data Analysis

The fMRI data were preprocessed using Data Processing and Analysis for Brain Imaging (DPABI)^1^ with the statistical parameter mapping software package (SPM12)^2^ and the Resting-State Functional MR imaging toolkit (REST)^3^ (Yan et al., 2016). First, the initial 10 volumes were discarded to stabilize the signal. Then, the remaining 200 volumes were realigned to the first volume after correcting for the differences in acquisition times, during which the mean frame-wise displacement (FD) was calculated. Data were excluded if head motion exceeded 2 mm and 2°. Two participants were excluded because of heavy head motion. The effects of nuisance signals and head motions (Friston-24 model) were also regressed out. Then, the diffeomorphic anatomical registration through exponentiated Lie algebra (DARTEL) tool was used for normalization (Asami et al., 2012), and the normalized data were finally band-pass filtered (0.01–0.08 Hz).

### Degree Centrality

The correlation matrix was obtained by calculating the Pearson correlation coefficient between the time course of one voxel within the predefined gray matrix mask and the time courses of all other voxels. Then, an undirected adjacency matrix was obtained by eliminating the weak correlation caused by noise through threshold processing of each correlation item at *r* > 0.25. Finally, *z*-score maps were obtained by converting the individual voxel-wise DC. The *z*-score maps were registered with 3-mm^3^ cubic voxels into the MNI space using the transformation information obtained from DARTEL and smoothed using a kernel of 6 mm.

### Statistical Analysis

Demographic data were analyzed using IBM SPSS Statistics (IBM SPSS Statistics for Windows, version 18.0, IBM Corp.). For detection of between-group differences in DC, the General Linear Model (GLM) with a paired *t*-test (resting wakefulness (RW) vs. SD) was used to identify regional DC changes. The threshold for significance was *P* < 0.05, corrected with the false discovery rate (FDR) criterion. The mean FD calculated during the preprocessing step was accounted for by including this term as a covariate. The differences between RW and SD were binarized as a mask for further machine learning analysis.

### Support Vector Machine Analysis

Trait-like individual differences in vulnerability to SD were defined using the same methods stated in our previous study (Zhu et al., 2017). Vulnerability to SD was computed on the basis of the extent of change in the number of lapses in each individual after SD. The participants were then ranked from highest to lowest per the vulnerability value. Finally, the participants were categorized into a vulnerability group and a resilience group.

The SVM was applied using the Pattern Recognition for Neuroimaging Toolbox (PRoNTo)^4^ to investigate whether the DC during RW could classify vulnerability to SD (Schrouff et al., 2013). In the first step (feature selection) the feature vector encoded the pattern of baseline DC values masked by the aforementioned mask. Feature selection comprised identifying brain regions that were expected to differ between the two sub-groups. These procedures were processed in the “Prepare feature set” program. In the second step, Leave-one-out cross-validation (LOOCV) was used to evaluate the performance of the classifier (Liu et al., 2020). In LOOCV, data from one subject was used as test data and the classifier is trained on the remaining dataset. These procedures were processed in the “Specify model” program. Next, once the SVM algorithm had been established, a 1,000-times permutation test was used to evaluate the performance of the SVM model. The corresponding accuracy, sensitivity, specificity, and area under the receiver operating characteristic curve were obtained. One advantage of the PRoNTo is that the weight map can be built at the voxel level. According to the contribution in the classification model, the region contributions can be ranked and presented for illustration. Finally, for each region, we used Pearson correlation to examine the associations between the changes in DC and PVT lapses using SPSS. Correction for multiple comparisons was accomplished using the FDR criterion (“mafdr” script implemented in MATLAB) (Zhu et al., 2019).

## Results

A total of 58 subjects successfully completed the SD experiment. Sleep diaries and Actiwatches confirmed that all subjects normally had good quality, habitual sleep. On the basis of the differences in the PVT lapses between the SD and RW conditions, subjects were divided into a vulnerable group and resilience group. The average number of PVT lapses for each group was 8.47 and 1.69, respectively. As expected, significant differences in PVT lapses were found between the two groups (*t* = 5.39, *p* < 0.001). No significant differences were found for gender, age, body mass index, or objective sleep measures observed via Actiwatches. Detailed sleep information is listed in Table 1.

**TABLE 1:** Demographic characteristics, objective sleep measures, and PVT performance.

	Vulnerable	Resilience	*p*-value
Gender (male/female)	15/14	15/14	1
Age (years)	22.4 ± 1.9	22.2 ± 1.6	0.43
Body mass index	23.7 ± 2.8	23.5 ± 2.3	0.81
**Objective sleep characteristics from Actiwatch**			
Time of falling asleep	00:05 ± 0:22	00:06 ± 0:27	0.86
Number of wakening each night	27.2 ± 6.4	27.4 ± 6.8	0.94
Sleep duration all night	6:45 ± 1:10	6:43 ± 1:25	0.91
Night sleep durations before work days	6:27 ± 0:52	6:25 ± 0:59	0.94
Night sleep durations before free days	7:06 ± 1:18	7:01 ± 1:19	0.83
Sleep efficiency in%	84 ± 2.8	83 ± 2.2	0.31
Sleep latency in minutes	16.6 ± 13.8	16.4 ± 14.3	0.84
**PVT performance**			
Number of lapse	8.47 (6.01)	1.69 (3.15)	<0.001

*Values represent mean ± SEM (*n* = 58); PVT, psychomotor vigilance task.*

**FIGURE 1:**
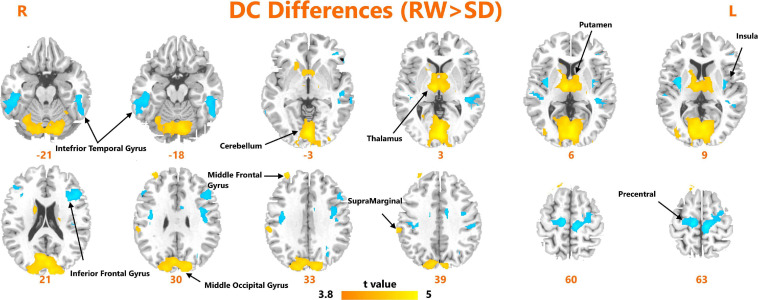
Areas of significant degree centrality differences between resting wakefulness state and sleep deprivation state.

A paired *t*-test was used to investigate the significant changes in DC measures after SD. As shown in Figure 1, we observed significantly increased DC within the bilateral inferior temporal gyrus, left insula, left inferior frontal gyrus, and bilateral precentral gyrus. We found significantly reduced DC within the bilateral cerebellum, thalamus, putamen, middle occipital gyrus, and right supramarginal gyrus.

**FIGURE 2:**
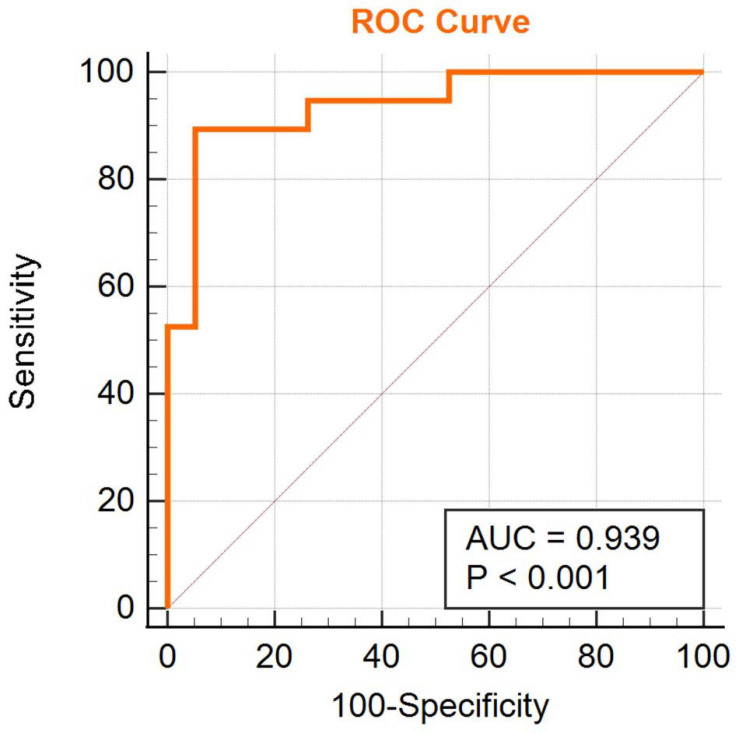
ROC curve of the classifier.

**FIGURE 3:**
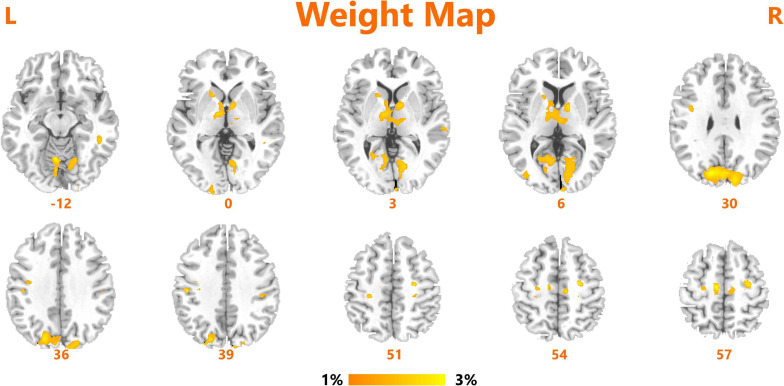
Brain regions of interest that contributed mostly to the accurate classification.

We obtained an accuracy of 84.75% with a sensitivity of 82.76% and specificity of 86.67% for classification of the two groups. The area under the curve was 0.939 (Figure 2). The brain regions that contributed most to the classification are shown in Figure 3 and listed in Table 2. The top 10 regions were the right supplementary motor area, right cerebellum, left inferior occipital gyrus, left precentral gyrus, left supramarginal gyrus, left thalamus, left middle temporal gyrus, left inferior parietal lobule, right middle frontal gyrus, and right middle occipital gyrus; their corresponding discriminative weights are also listed in Table 2.

**TABLE 2:** The top ten ranked regions that contributed mostly to the classification.

Brain regions	Cluster size	Peak coordinates (MNI)			Discriminative weight (%)
		*X*	*Y*	*Z*	
Supplementary motor area R	290	9	−15	57	5.02
Cerebellum R	1402	6	−63	−12	3.84
Inferior occipital gyrus L	46	−21	−99	−12	3.81
Precentral gyrus L	80	−24	−12	51	3.53
Supramarginal R	92	48	−24	36	2.98
Thalamus L	403	−12	−3	6	2.58
Middle temporal gyrus L	88	−63	−27	0	2.44
Inferior parietal lobule L	21	−42	−30	39	2.31
Middle frontal gyrus R	21	24	−21	54	2.22
Middle occipital gyrus R	92	36	−84	3	1.85

**FIGURE 4:**
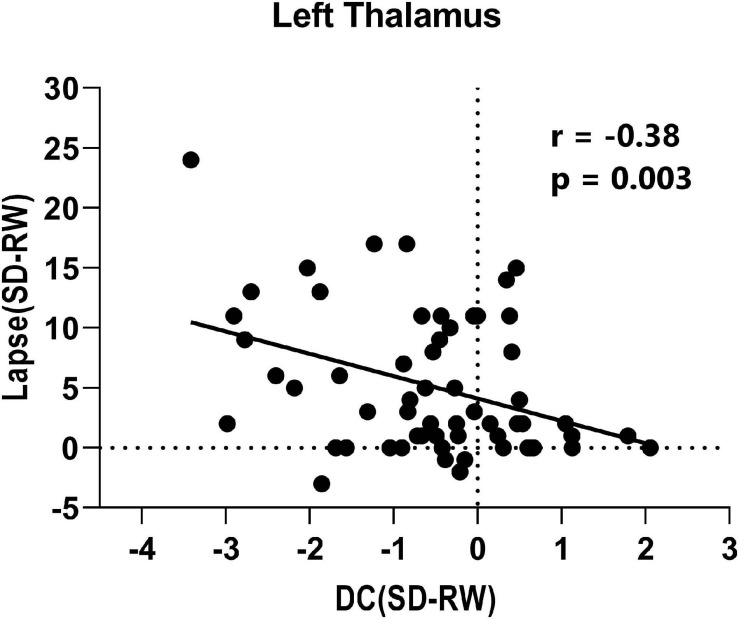
Correlation between change of lapse of psychomotor vigilance task and change of degree centrality within left thalamus.

Finally, the mean DC changes (SD-RW) within each region were extracted and plotted against the changes in PVT lapses. We found a significantly negative correlation with the left thalamus (see Figure 4).

## Discussion

Using a multivariate pattern classification method, the present study demonstrates that degree centrality derived from fMRI data collected during RW can be used to classify subjects on the basis of whether they are vulnerable or resilient to SD. With excellent accuracy, the brain regions that showed the most discriminatory power were mainly located within the sensorimotor network (SMN), DMN, and thalamus. Furthermore, we found a significant negative correlation between the changes in PVT lapses and DC in the thalamus after SD. These findings suggest that graph-theory-based measures, such as DC, combined with machine-learning algorithms, can help to predict vulnerability to SD.

Because SD has become very common in contemporary 24/7 society, efficient screening for resilient and vulnerable people has social significance. Although previous studies have used baseline measures of psychomotor vigilance and the drift diffusion model to classify vulnerability to SD (Patanaik et al., 2014), the accurate classification rate was around 77–82%, which was less than satisfactory. Vulnerability to SD has been shown to be stable and trait-like, with characteristic neural correlates that have been identified. Therefore, neuroimaging data combined with state-of-the-art artificial intelligence algorithms might enable greater classification performance. Our results verified that SD leads to significant DC reductions in the cerebellum, thalamus, and putamen. This indicates that functional connections within subcortical regions are compromised, which is consistent with previous studies (Nechifor et al., 2020). A significant increase in DC was mainly found within the SMN and DMN, which suggests that SD affects lower functional network segregation and higher network integration (Yu et al., 2017).

The brain regions that contributed most to the classification model include the supplementary motor area, middle temporal gyrus, and middle frontal gyrus, which are core regions of the DMN. The DMN is more active during passive tasks than during externally orientated tasks, and has been extensively examined in SD research (Gujar et al., 2010). Furthermore, the anti-correlation between sub-networks of the DMN and frontal-parietal networks subserves working memory performance during the mid-point of night in the regular biological sleep cycle (Zhu et al., 2019). These consistent findings highlight the role of the DMN in predicting vulnerability to SD.

Apart from that related to the DMN, another interesting finding of the present study is that the thalamus also exerts an important role in modulating SD vulnerability. The thalamus is one of the core network brain regions that subserves vigilant attention in humans (Avanzini et al., 2000). Previous studies have indicated that the thalamus is involved in sensory gating and attentional modulation by acting as a bridge between sensory perception and cognition (Saalmann and Kastner, 2011). Increased thalamus activation has been frequently reported in SD studies (Hershey et al., 1991; Gent et al., 2018). However, the activity pattern in the thalamus has been found to be correlated significantly with mean melatonin levels, and therefore, the thalamus is modulated more by circadian rhythms than by sleep debt (Muto et al., 2016; Zhu et al., 2020). Previous studies have indicated that SD vulnerability is stable after total SD or short periods of sleep restriction, suggesting that SD vulnerability is not solely modulated by sleep debt (Van Dongen et al., 2004; Rupp et al., 2012). The common patterns found in thalamus activity and vulnerability to SD, coupled with the discriminative weight and negative correlation found in the current study, imply that baseline activity within the thalamus has broad potential applications in screening for SD vulnerability.

Several limitations are present in the current study. First, the sample size was relatively small. However, we selected the SVM algorithm for classification because it has good efficiency when used with small sample sizes. Second, although it is possible that micro-sleep occurred during the SD period, two research assistants were present to prevent subjects from falling asleep, so this is unlikely. Furthermore, the subjects were required to stay awake and keep their eyes open during the scanning procedure, and their heart rate and breathing frequency were collected concurrently to verify that they were not asleep.

## Conclusion

Our study demonstrates that graph-theory-based DC measures combined with machine learning algorithms have the potential to predict vulnerability to SD. Brain regions within the SMN, DMN, and thalamus contributed most to the accurate classification model. Future studies may benefit from the integration of white matter connectivity or other imaging modality measurements.

## Data Availability Statement

The raw data supporting the conclusions of this article will be made available by the authors, without undue reservation.

## Ethics Statement

The studies involving human participants were reviewed and approved by Committee of Xijing Hospital at the Air Force Medical University. The patients/participants provided their written informed consent to participate in this study.

## Author Contributions

YX, PY, and JZ performed all data analysis and wrote the manuscript. YZ raised the conception of the study. XT, CW, and FR contributed to the collection of MRI data. ZX, TH, QY, and FG contributed to the manuscript revision. All authors read and approved the submitted version.

## Conflict of Interest

The authors declare that the research was conducted in the absence of any commercial or financial relationships that could be construed as a potential conflict of interest.

## Footnotes

1
http://rfmri.org/dpabi

2
https://www.fil.ion.ucl.ac.uk/spm/

3
http://www.restfmri.net

4
http://www.mlnl.cs.ucl.ac.uk/pronto
