# Corrigendum: Classifying Vulnerability to Sleep Deprivation Using Resting-State Functional MRI Graph Theory Metrics

**DOI:** 10.3389/fnins.2022.923418

**Published:** 2022-06-24

**Authors:** Yongqiang Xu, Ping Yu, Jianmin Zheng, Chen Wang, Tian Hu, Qi Yang, Ziliang Xu, Fan Guo, Xing Tang, Fang Ren, Yuanqiang Zhu

**Affiliations:** ^1^Department of Radiology, Xijing Hospital, Fourth Military Medical University, Xi'an, China; ^2^Affiliated Wuhan Mental Health Center, Tongji Medical College, Huazhong University of Science and Technology, Wuhan, China; ^3^Department of Radiology, Yan'an University Affiliated Hospital, Yan'an, China; ^4^Department of Radiology, Affiliated Hospital of Shaanxi University of Traditional Chinese Medicine, Xianyang, China

**Keywords:** sleep deprivation, vulnerability, functional magnetic resonance imaging, machine learning, psychomotor vigilance task

“Minwen Zheng” was mistakenly included as an author in the published article. The contribution of this author was removed from the published article. The corrected “Author Contributions” statement appears below.

## Author Contributions

YX, PY, and JZ performed all data analysis and wrote the manuscript. YZ raised the conception of the study. XT, CW, and FR contributed to the collection of MRI data. ZX, TH, QY, and FG contributed to the manuscript revision. All authors read and approved the submitted version.

The authors apologize for this error and state that this does not change the scientific conclusions of the article in any way. The original article has been updated.

## Publisher's Note

All claims expressed in this article are solely those of the authors and do not necessarily represent those of their affiliated organizations, or those of the publisher, the editors and the reviewers. Any product that may be evaluated in this article, or claim that may be made by its manufacturer, is not guaranteed or endorsed by the publisher.

